# Vaginal Microbiome in Pregnant Women with and without Short Cervix

**DOI:** 10.3390/nu15092173

**Published:** 2023-05-02

**Authors:** Angela Silvano, Niccolò Meriggi, Sonia Renzi, Viola Seravalli, Maria Gabriella Torcia, Duccio Cavalieri, Mariarosaria Di Tommaso

**Affiliations:** 1 Department of Health Sciences, Division of Obstetrics and Gynecology, Careggi Hospital, 50139 Florence, Italy; angela.silvano@unifi.it (A.S.); viola.seravalli@unifi.it (V.S.); 2 Department of Biology, University of Florence, Via Madonna del Piano 6, 50019 Florence, Italy; niccolo.meriggi@unifi.it (N.M.); sonia.renzi@unifi.it (S.R.); 3 Department of Clinical and Experimental Medicine, University of Firenze, 50139 Firenze, Italy; maria.torcia@unifi.it

**Keywords:** shortened cervix, *Lactobacillus*, *Gardenerella vaginalis*, aerobic vaginitis, microbiome, risk in pregnancy

## Abstract

Cervical shortening is a recognised risk factor for pre-term birth. The vaginal microbiome plays an essential role in pregnancy and in maternal and foetal outcomes. We studied the vaginal microbiome in 68 women with singleton gestation and a cervical length ≤25 mm and in 29 pregnant women with a cervix >25 mm in the second or early third trimester. Illumina protocol 16S Metagenomic Sequencing Library Preparation was used to detail amplified 16SrRNA gene. Statistical analyses were performed in R environment. *Firmicutes* was the phylum most represented in all pregnant women. The mean relative abundance of *Proteobacteria* and *Actinobacteriota* was higher in women with a short cervix. Bacterial abundance was higher in women with a normal length cervix compared to the group of women with a short cervix. Nonetheless, a significant enrichment in bacterial taxa poorly represented in vaginal microbiome was observed in the group of women with a short cervix. *Staphylococcus* and *Pseudomonas,* taxa usually found in aerobic vaginitis, were more common in women with a short cervix compared with the control group, while *Lactobacillus iners* and *Bifidobacterium* were associated with a normal cervical length. *Lactobacillus jensenii* and *Gardenerella vaginalis* were associated with a short cervix.

## 1. Introduction

Vaginal microbial communities play a significant role in the health of mother and foetus and in the maintenance of a favourable microenvironment during pregnancy [1,2,3]. The composition and diversity of vaginal microbiota are modified in pregnancy [4,5]; indeed, microbiota has proved to be less rich and varied in pregnant women compared to nonpregnant women [6,7]. Diversity of the vaginal microbiome profile also depends on the race and ethnicity of pregnant [8] and non-pregnant women of reproductive-age [9]. Vaginal microbiota is dominated by *Lactobacillus* species in the majority of women and *Lactobacillus* spp. is maintained throughout the entire gestation in healthy pregnancies with normal outcomes [10].

The microbial communities referred to as community state types (CSTs), are clustered into five groups, of which four are dominated by *Lactobacillus* [9]. CST-I is dominated by *Lactobacillus crispatus*, CST-II by *L. gasseri*, CST-III by *L. iners*, CST-V by *L. jensenii* and CST-IV is represented by polymicrobial communities that include bacteria and higher proportions of strictly anaerobic organisms belonging to *Gardnerella*, *Atopobium*, *Mobiluncus*, *Megasphoera*, *Prevotella*, *Streptococcus*, *Mycoplasma*, *Ureaplasma*, *Dialister* and *Bacteroides* genera [11,12,13]. Several studies have attempted to link vaginal microbiota to preterm birth [1,10,14,15]. The risk of spontaneous preterm birth is associated with a short cervix [16,17], and a variety of etiopathogenetic mechanisms may be involved including uterine overdistension [18] and changes in the cervico-vaginal microbiome, among others [19].

Indeed, it has been demonstrated that *Lactobacillus crispatus* dominance is protective against preterm birth [20,21]. In contrast, a significant increase in the richness and diversity of the vaginal microbial community is considered to be a risk factor for preterm birth [22]. 

In this study we aimed to describe the vaginal microbial populations across the second or early third trimester in pregnant women of predominantly Caucasian ethnicity with and without a short cervix and to compare the composition, diversity and evenness of the vaginal bacterial communities.

## 2. Materials and Methods

### 2.1. Study Design

The study was conducted between 2015 and 2021 at the Department of Obstetrics and Gynecology of Careggi University Hospital in Florence, Italy. Sixty-eight women with singleton gestation and a cervical length ≤ 25 mm (cases) and twenty-nine pregnant women with a cervix > 25 mm (controls) in the second or early third trimester (23–32 weeks’ gestation) were prospectively enrolled. Women with a history of preterm birth, prior surgery to the cervix, evidence of premature rupture of membranes, symptomatic uterine contractions, antibiotic treatment or vaginal symptoms consistent with infection at the time of recruitment were excluded. The presence of a cervical cerclage or pessary, and a diagnosis of fetal malformation, were also considered within the exclusion criteria. 

Ethics approval for this study was granted by the Institutional Ethics Committee (Ref. no. BIO14.0009-09/07/2014), and all women provided written informed consent. The cases were referred to the Hospital’s Preterm Birth Clinic by their obstetricians who detected cervical shortening on a transvaginal ultrasound performed during a routine prenatal visit. Although not under a specific protocol, in private practice in Italy pregnant women are often offered cervical length measurement and this can lead to a diagnosis of a short cervix even after 24 weeks of gestation. Controls were selected among women attending the Hospital’s Maternity Outpatient Clinic for routine prenatal visits.

### 2.2. Sample Collection and DNA Extraction

Vaginal secretions were collected by inserting a swab approximately 4 to 5 cm into the vagina and gently rotating it several times. The swab was then placed in phosphate buffer saline on ice for 30 min. After swab removal, samples were centrifuged at 8000× *g* for 10 min; the pellet was stored immediately at −80 °C.

Total DNA was extracted from the pellet using the DNeasy PowerSoil Kit (Qiagen, Hiledn, Germany) according to the manufacturer’s instructions. DNA quality and integrity were checked on 1% agarose gel and quantified using the Qubit 4 Fluorometer (Thermo Fisher Scientific, Waltham, MA, USA) with the 1× dsDNA High Sensitivity kit.

### 2.3. Library Preparation and Sequencing

For each DNA sample, the bacterial 16S rRNA gene was amplified using a primer set specific for the V3–V4 hypervariable regions (341f: 5′-CCTACGGGNGGCWGCAG-3′ and 805r: 5′-GACTACNVGGGTWTCTAATCC-3′) [23], provided with overhang Illumina adapters. Libraries have been prepared following Illumina protocol 16S Metagenomic Sequencing Library Preparation (Part # 15044223 Rev. B, 2013). Paired end 2 × 300 bp sequencing was performed on an Illumina MiSeq instrument (Illumina Inc., San Diego, CA, USA), using MiSeq Reagent Kit v3 (600 cycle), at the Department of Biology, University of Florence, Italy.

### 2.4. Amplicon Sequence Variance Assemblage

Primer pair sequences were removed by using cutadapt version 1.15 [24] in paired-end mode. If no primer sequence was found, the entire sequence was discarded together with its pair to reduce possible contamination. The raw sequences were processed using DADA2 pipeline version 1.26.0 [25] to infer amplicon sequence variants (ASVs). The “filterAndTrim” function was performed to filter low quality sequences with a maximum number of expected error thresholds of 2 for forward and reverse read pairs. The error rate estimation using the “learnErrors” function and denoising using the “dada” function with default parameters were performed. Denoised reads were merged using the “mergePairs” function, discarding those with any mismatches and/or an overlap length shorter than 12bp. Chimeric sequences were removed using the “removeBimeraDenovo” function and taxonomic classification was produced by using DECIPHER package version 2.14.0 against the pre-formatted Silva small-subunit reference database [26] SSU version 138 available at: http://www2.decipher.codes/Downloads.html (accessed on 2 March 2023). All sequence variants not assigned to bacteria (unknown) or assigned to chloroplasts and mitochondria sequences were removed from the dataset to properly perform the downstream statistical analyses.

### 2.5. 16 S Metabarcoding Statistical Analysis

Statistical analyses were performed in R environment version 4.1.2 (R Core Team, 2021). Mean relative abundance was calculated by using the “microbiomeutilities” package version 1.0.16 [27]. Differences in bacterial diversity (beta diversity analysis) were inspected using the “vegan” package version 2.6.2 [28]. In detail, sample distribution was displayed by Non-metric multidimensional scaling (NMDS) using the “metaMDS” function of the “vegan” package version 2.6.2 performed on distance matrices based on the Bray–Curtis dissimilarity index. Before multidimensional analysis, counts present less than 1 time within the sample dataset were removed, then all counts were transformed using relative abundance transformation to reduce coverage bias among samples. Permutational multivariate analysis of variance using distance matrices (adonis permanova) was performed to inspect differences between sample groups by using the “adonis2” function of the “vegan” package version 2.6.2. Adonis permanova was tested on multiple-factor formula which included the variables Group, Gestational diabetes, Cervicometry group, Preterm delivery and Progesterone therapy. Adonis permanova was carried out using 1000 numbers of permutations. Group refers to group division based on cervix length ≤ 25 or >25 mm, while Cervicometry group refers to the groups produced after the division based on three cervix lengths, that is 0–10, 11–24 and **≥**25 mm. Alpha diversity measures (Observed and Shannon index) were produced by the “estimate_richness” function from phyloseq package version 1.42 [29] while Evennes was defined as the Shannon diversity index/log(observed richness) [30]. Significant effects were inspected using the pairwise Wilcoxon test by using the “wilcox_test” function from the “rstatix” package version 0.7.0, adjusting resulting *p*-values with the Benjamini–Hochberg correction method [31]. Correlation between alpha metrics and cervix length was produced by the “stat_cor” function of the “ggpubr” package version 0.4.0 [32].

To detect statistically significant taxonomic features we performed a linear discriminant analysis effect size (LEfSe) test after counts per million (CPM) transformation [33]. LEfSe was conducted by using Galaxy implementation (https://huttenhower.sph.harvard.edu/galaxy/ accessed on 2 March 2023), setting the Group variable as class and samples as subject. Alpha value 0.05 was considered for both the factorial Kruskal–Wallis test among classes and for the pairwise Wilcoxon test between subclasses. One-against-all as a strategy for multi-class analysis was conducted, and 2 was set as the logarithmic LDA score threshold for discriminative features. Differential abundance analysis was performed using the Wald test (*p*-value from Wald test adjusted for multiple testing using the Benjamini and Hochberg method) implemented in the DESeq2 package version 1.38.3 [34]. Before the Wald test, the singletons were removed to dampen the hypothesis that extremely rare species could be considered major drivers of differences in groups. The size factor estimation was computed using the “postcount” method to estimate ASVs geometric means in the presence of zeros by the “estimateSizeFactors” function of the “DESeq2” package. Sequence variants’ dispersion was fitted using local regression by the “estimateDispersions” function with the “local” fitting method of the “DESeq2” package. Figures were produced using the “ggplot2” package version 3.4.1 [35] and edited using open-source graphics editor Inkscape (http://inkscape.org/ accessed on 6 March 2023).

## 3. Results

### 3.1. Population Characteristics

The demographic and clinical characteristics of the enrolled patients are summarised in Table 1. Most patients (98%) were of Caucasian ethnicity. The mean gestational age at sampling was 28.4 weeks, and did not differ between cases and controls (Table 1). The mean cervical length was 15 mm in the case group and 32 mm in the control group. Supplementary clinical information were also considered; in particular, the selected subjects did not present differences in the weight of the new-born children (Kruskal–Wallis test, *p* = 0.77). Moreover, subjects who were undergoing probiotic therapy did not present differences in cervix length (Anova, *p* = 0.32).

### 3.2. Vaginal Microbial Community Composition and Diversity

**Figure 1:**
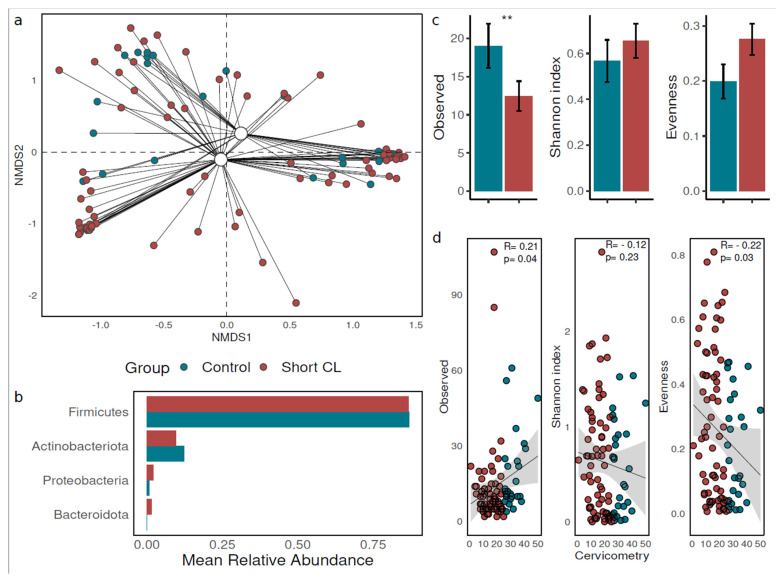
Bacterial diversity among groups with different cervix length. (**a**) Non-metric multidimensional scaling (NMDS) analysis based on Bray–Curtis distance showing the distribution of samples with a short cervix length and controls (Group). Samples are represented using solid-coloured points while white-filled points represent centroids. (**b**) Barplot showing the Phylum-level mean relative abundance. The mean relative abundance measure reports the taxa present in more than 10% of samples within the dataset. (**c**) Barplot reporting the alpha diversity measures represented by total number of ASVs (Observed), Shannon index and Evenness (*y*-axis) among groups with and without short cervix (control group). Asterisks are shown above the barplot to indicate a significant effect after Kruskal–Wallis test comparison (**, *p*-value < 0.01). (**d**) Scatterplot showing the correlation between alpha diversity measures (Observed, Shannon index and Evenness) on *y*-axis and cervical length (Cervicometry) expressed in mm on *x*-axis. The shaded grey region around the linear regression line represents 95% confidence intervals. Results from linear regression analysis calculated using Spearman’s correlation method are reported inside the regression panels. Samples and sample groups in each panel were depicted according to the colour pattern in the legend.

A DADA2 pipeline produced a total of 5,138,086 reads clustered in a total of 742 different ASVs inferred from 16S metabarcoding sequencing. After quality filtering (removal of unknown, chloroplasts and mitochondria sequences) a total of 5,091,464 reads clustered in 476 different bacterial sequence variants were obtained. The dataset was widely represented by the taxa belonging to the phylum *Firmicutes* with a mean relative abundance of 87% (Table S2 and Figure 1b). Taxa belonging to the phylum *Actinobacteriota* and *Proteobacteria* were also represented with mean relative abundance of 12% and 1.1% respectively (Table S2 and Figure 1b). Phyla with mean relative abundance lower than 1% of the entire abundance within the dataset were also highlighted (Table S2). 

Non-metric multidimensional scaling (NMDS) analysis based on Bray–Curtis distance among samples with different cervix length were performed. Ordination analysis showed substantial overlap among sample groups (Group in Figure 1a). This evidence was confirmed by the permutational multivariate analysis of variance based on the Bray–Curtis distance matrix (adonis permanova) which does not show a significant effect of the cervix length variable (Group in Table S1) or of the other factors included in the model formula (Table S1). Thus, no factors tested influence bacterial diversity (referred as beta diversity). The alpha diversity analysis showed no significant differences in the Shannon index and Evenness diversity measures between sample groups with different cervix length (Kruskal–Wallis; Shannon index, *p* = 0.73 and Evenness, *p* = 0.18) (Figure 1c), but a significant difference in the total number of ASVs was depicted (Kruskal–Wallis; Observed, *p* < 0.01) (Figure 1c). In particular, control subjects showed a significantly higher number of ASVs than subjects with short cervix length (Figure 1c). Correlation analysis between alpha diversity measures and cervix length were also tested. Significant positive correlation between the total number of ASVs and cervix length was highlighted, thus demonstrating an increase in bacterial abundance as the length of the cervix increases (Figure 1d). An overall negative correlation among diversity measures (Shannon index and Evenness) and cervix length was observed. In detail, no significant correlation was obtained for the Shannon index but a significant result was observed for the evenness index (Figure 1d), highlighting a progressive decrease in bacterial homogeneity as the length of the cervix increases. 

### 3.3. Bacterial Biomarkers

**Figure 2:**
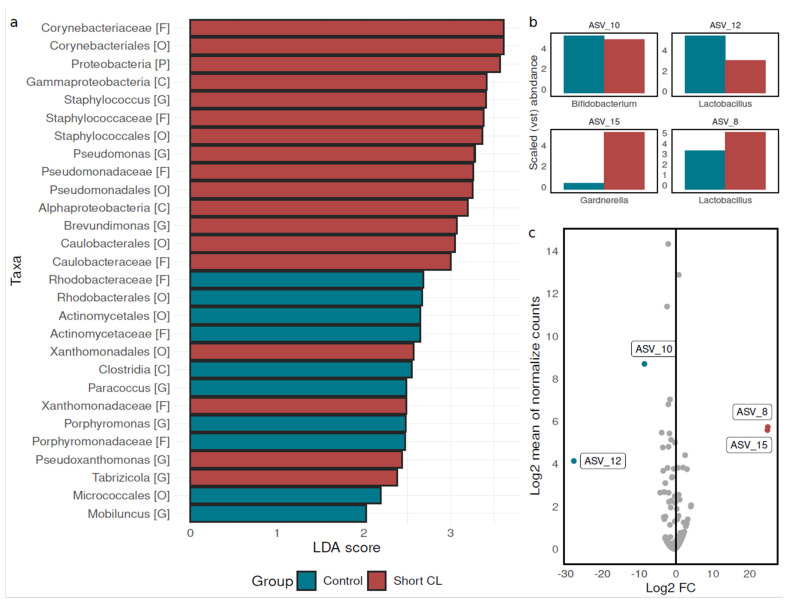
Significant bacterial features among groups with different cervix length. (**a**) Linear discriminant analysis effect size (LEfSe) showing the significant taxonomic features between short cervix length and control groups at different taxonomic levels. Linear Discriminant Analysis scores (LDA score) are reported on *x*-axis (LDA threshold > 2) while taxa at different taxonomy levels are reported on *y*-axis. (**b**) Variance stabilizing transformation (VST) abundance after DESeq postcount transformation depicted for the four significant ASV obtained after Wald test. On the bottom chart the ASV taxonomic assignment was also reported and the abundance was reported for the group with a short cervix length and the control group according to the colour pattern in the legend. (**c**) MA plot reports the base 2 logarithm of normalised counts across all samples vs. the base 2 logarithm of fold-change (Log2FC). ASVs with fdr <0.05 are depicted using the cervix length group’s colour pattern while the others are reported in light grey.

Linear discriminant analysis effect size (LEfSe) testing detected 28 different taxonomic features significantly related to the group with a short cervix length and the control group (Figure 2a). Five genus-level taxonomic features were significantly associated with groups with a short cervix length; in detail the genera *Staphylococcus*, *Pseudomonas*, *Brevundimonas*, *Pseudoxanthomonas*, *Tabrizicola* were depicted (Figure 2a). Therefore, the genera *Paracoccus*, *Porphyromonas* and *Mobiluncus* were significantly highlighted in the control group (Figure 2a). In order to deepen the taxonomic characterisation between the two groups, a ASV-level differential expression analysis approach was taken. Therefore, to detect the bacterial ASVs which significantly change within the two sample categories (Groups) we performed a Wald test implemented in the DESeq2 package. The Wald test detected 4 ASVs differentially enriched among the short cervix group and control group. Significant ASVs corresponded to 0.85% of the total number of ASVs within the dataset and accounted for 3.75% of the entire ASVs abundance (mean in each sample 7.12% and standard error 2.11%). The differences between groups with different cervical length showed that ASV_10 and ASV_12, assigned to genera *Bifidobacterium* and *Lactobacillus,* respectively, were significantly enriched in the control group, while ASV_8 and ASV_15, assigned to the genera *Lactobacillus* and *Gardnerella,* respectively, were associated with the short cervix length group (Figure 2b). The ratio between the ASVs base 2 logarithm of normalised counts across all samples on the base 2 logarithm of fold-change highlighted for the ASVs tested using DESeq2 was depicted in Figure 2c.

Since ASV_12 and ASV_8 were both assigned to the genus *Lactobacillus,* we performed the alignment of the nucleotide sequence of the related ASVs with nucleotide BLAST, and thus we identified the following taxonomic species, *Lactobacillus jensenii* (ASV_8) and *Lactobacillus iners* (ASV_12), sorted on the basis of the best similarity score (nucleotide sequences were reported in Table S4 and alignment output was reported in Table S3). The first best result obtained from BLAST alignment provided an identity score > 99%, for each sequence aligned (Table S3). 

## 4. Discussion

The cervix plays an essential role in pregnancy, protecting the developing foetus and preventing infection ascending from the vaginal canal to the uterine cavity. Indeed, infection and local inflammation in the cervix may compromise cervical integrity and result in premature remodelling, predisposing to spontaneous preterm birth [36]. Cervicovaginal microbiota play a key role in pregnancy outcome. A vaginal microbiota not dominated by *Lactobacillus* species affects the physiological wellbeing of the cervix and increases the risk of ascendent infections and spontaneous preterm birth [10,22,37].

In our study, pregnant women with and without cervical shortening were enrolled and their vaginal fluid was analysed. Some of the women enrolled were treated with vaginal progesterone for its anti-inflammatory and pro-relaxant activity in the uterus [38]. This treatment, however, has been repeatedly shown not to affect the composition of vaginal microbiota [20].

According to previously reported data, *Firmicutes* was the phylum most represented in the two groups [21]. Increased mean relative abundance in *Proteobacteria* and *Actinobacteriota,* however, was evident in the group of women with a short cervix. Other factors such as diabetes mellitus and progesterone vaginal therapy did not affect bacterial diversity either.

Women with a normal cervical length showed a higher number of ASVs compared to women with a shorter cervical length, suggesting that the overall amount of bacterial microbiota is affected by pathological conditions that lead to cervical shortness. Agreeing with data reported in the literature [37], the Evenness index and cervical length were negatively correlated, suggesting an increase in bacterial diversity as the length of the cervix decreases.

Significant enrichment in bacterial taxa were revealed by LEfSE analysis in pregnant women with a short cervix compared to controls. In particular, we observed significant enrichment in *proteobacteria*, a phylum including a high number of pathobionts, in women with a short cervical length. At genus level, a significant enrichment in *Staphylococcus* and *Pseudomonas* genera was observed in the vaginal microbiota of women with a short cervix. These genera include many proinflammatory bacterial species that are usually found in aerobic vaginitis, [39]. Moreover, *Brevundimonas*, a genus of non-fermenting Gram-negative bacteria that has recently been considered to be an emerging opportunistic pathogen [40], was also significantly enriched in women with a short cervix.

Aerobic vaginitis is a vaginal condition, distinct from bacterial vaginosis, which is characterised by extreme inflammatory changes and desquamative vaginitis [41]. It is associated with increased risk for preterm delivery [42] and, recently, the pathogenic properties of bacterial taxa associated with aerobic vaginitis have been described in women with recurrent spontaneous abortions [43]. *Pseudomonas species*, moreover, have also been associated with recurrent miscarriages [44].

Thus, the enrichment in *Staphylococcus* and *Pseudomonas* genera observed in the vaginal microbiota of women with a short cervix may have clinical relevance in the risk assessment for a short cervix and pre-term birth [21,45].

The Wald test (implemented in the DESeq2 package), identified 4 ASVs differentially enriched in the two groups of pregnant women and the alignment of the nucleotide sequence of enriched ASVs with nucleotide BLAST revealed that *Lactobacillus jensenii* and *Gardnerella vaginalis* were associated with pregnant women with a short cervix length.

*Lactobacillus iners* and *Bifidobacterium* were associated with a normal cervical length. The data on the association of *Gardnerella species* with a short cervix are in agreement with the data reported by Witkins SS [21,46]. *Gardnerella vaginalis* is a bacterial species which is usually dominant in bacterial vaginosis. Selected strains of *Gardnerella vaginalis* produce a huge amount of sialidase, an enzyme that digests the mucus protection of vaginal epithelium allowing bacteria to adhere to epithelial cells following displacement of lactobacilli. *Gardnerella vaginalis* is also the bacterial species involved in the biofilm formation that allows the enrichment of aerobic and anaerobic species in the vaginal microenvironment [13].

The differential expression of *Lactobacillus jensenii* in women with a short cervix was not expected. *Lactobacillus jensenii* is a *Lactobacillus* species dominant in the vaginal microbiota of 5% of women in reproductive age [9]. This *Lactobacillus* species was reported as being equally protective as *Lactobacillus crispatus* against infections with pathogenic bacteria, particularly for its high production of D-lactic acid [47]. *Lactobacillus jensenii* also induces high production of IFN-gamma and IL-10 by cells of the immune system [48] and contributes to balancing immune reactions against invading pathogens. Our data, however, confirm the microbial signatures published by Pausan et al., 2020, that associate *Lactobacillus jensenii*, *Lactobacillus gasseri*, *Ureaplasma* sp. and *Gardnerella* sp. with a short cervix length, PTB and/or preterm contractions [49]. 

Further investigations are needed to understand the role of *Lactobacillus jensenii* in events leading to cervical shortening. 

The protective mechanisms associated with *Lactobacillus* spp. have been widely described in the literature [50]. *Lactobacillus* spp. predominates in low pH environments; vaginal acidity prevents colonization by anaerobes, maintains the cervical epithelial barrier through production of bacteriocins, and acts against mucin degradation, keeping away opportunistic infections. In addition to lactic acid, other substances such as hydrogen peroxide are produced by lactobacilli in the vaginal microenvironment to inhibit the growth of potential pathogens [51]. Additionally, *Bifidobacteria* have health-promoting effects in the vaginal environment and a probiotic potential; they promote immune modulation, production of bacteriocins and inhibition of pathogens [6], and have a beneficial effect on the vaginal environment. In our study, *Bifidobacterium* was highly represented in pregnant women with a normal cervical length (>25 mm), suggesting a possible role in the control of harmful bacterial species. Furthermore, *Lactobacillus iners* was also observed to be enriched in the group of women with a normal cervical length in our study, thus further defining the association and the possible role of lactobacilli in the vaginal microbiota. A significant positive association was found between *Lactobacillus iners* and the occurrence of spontaneous preterm birth in a cohort of predominantly Caucasian and Asian women with a short cervix [20] as well as in women of Caucasian ethnicity with an extremely short cervix (<10 mm) [21]. However, an exploratory study of associations with spontaneous preterm birth in pregnant women with a normal cervical length [52] demonstrated that *Lactobacillus iners,* which is a dominant species in the vaginal microbiota, was associated with an increased occurrence of spontaneous preterm birth, regardless of cervix length. Therefore, increasing the number of pregnant women to be enrolled in microbiome evaluation studies is essential to clarify the role of *Lactobacillus iners* as a marker of cervical shortening. 

Our results further define the contribution of vaginal microbiota in pregnant women with a short cervical length. We have highlighted how the total abundance of taxonomic variants is correlated with cervical length and we have also described the presence of taxonomic variants associated with a short cervix; therefore it is not unexpected that these may play a role within the vaginal microbial ecology. Some of the identified communities were in agreement with the results of previous studies, while others were described for the first time as markers of association with cervical length, or were unconfirmed as markers of association with cervical length. Therefore, further studies are needed to determine whether the microbial signature reported in the present study is confirmed in a larger cohort of women. Overall, our data highlight an association between cervical length and vaginal bacterial communities, and this further defines the importance of studying the vaginal microbiota to understand the associated clinical conditions.

## Figures and Tables

**Table 1:** Patients’ demographic and clinical characteristics.

	All Women	Cases with Cervical Length ≤ 25 mm	Controls with Cervical Length > 25 mm	*p*-Value
Number of enrolled pregnant women	97	68	29	/
Ethnicity *n* (%) Caucasian Asian North-African	95 (98%) 1 (1%) 1 (1%)	66 (97%) 1 (1.4%) 1(1.4%)	29 (100%) 0 0	/
Body mass index mean ± SD	23.6 ± 5.4	23.0 ± 4.97	24.9 ± 6.1	0.16
Smoking * n/nTot (%)	8/91 (8.8%)	7/68 (10.3%)	1/23 (4.3%)	0.67
Age at sampling (years) mean ± SD	33.8 ± 6.4	33.7 ± 6.4	34.0 ± 6.4	0.993
Gestational age at sampling (weeks) mean ± SD	28.4 ± 2.6	28.2 ± 2.5	28.6 ± 3.0	0.775
Length of the cervix at sampling (mm) mean ± SD	20.0 ± 9.5	15.1 ± 5.8	31.6 ± 5.7	<0.001 ***
Gestational diabetes mellitus *n* (%)	27 (27.8%)	17 (25%)	10 (14.7%)	0.352
Gestational age at delivery (weeks) mean ± SD	37.6 ± 2.9	37.5 ± 2.7	37.6 ± 3.3	0.145

Demographic and clinical information on the enrolled patients was reported with *p*-value after a group comparison Fisher’s Exact test was performed for smoking frequency comparison, a Kruskal–Wallis test was performed for body mass index comparison and an Anova test was performed for all other comparisons. * Missing data for 6 subjects. *** *p*-value < 0.001.

## Data Availability

The 16S rRNA raw sequences have been deposited to the European Nucleotide Archive (ENA) under the accession code PRJEB60394. All results from statistical analyses were mentioned by writing in the main text, reported as figures or tables in the main text and supplementary materials.

## Supplementary Materials

The following supporting information can be downloaded at: https://www.mdpi.com/article/10.3390/nu15092173/s1, Table S1: Results from permutational multivariate analysis of variance (adonis permanova) based on Bray-Curtis distance matrix. Table S2: Phylum-level abundance summary. Table S3: Results form nucleotide BLAST alignment. Table S4: DNA sequences aligned by using nucleotide BLAST. Table S5: Results from DESeq analysis.
