# Supplementary material for: Vaginal Microbiome in Pregnant Women with and without Short Cervix
Source: Nutrients. 2023 May 2;15(9):2173. doi: 10.3390/nu15092173 (PMC10180705; doi:10.3390/nu15092173)
Supplement: Supplementary file 1 [file nutrients-15-02173-s001.zip › Supplementary materials Silvano et., 2023 Nutrients.pdf]

Supplementary materials

Supplementary table S1. Results from permutational multivariate analysis of variance (adonis permanova) based on Bray-Curtis distance matrix. Variance explained (R2 values) and Pr(>F) were reported for each factor present in the tested formula (Factor). The proportion of variance unexplained by the factor considered was reported in the Residual column.

**Factor**	**Df**	**SumOfSqs**	**R2**	**F**	**Pr(>F)**
Group	1	0.561	0.015	1.476	0.162
Gestational diabetes	1	0.218	0.006	0.567	0.758
Cevicometry group	2	0.681	0.019	0.885	0.616
Preterm delivery	2	0.931	0.025	1.210	0.262
Progesterone therapy	1	0.504	0.014	1.310	0.218
Residual	88	33.875	0.921		
Total	95	36.771	1		

Supplementary table S2. Phylum-level abundance summary. Table reported the maximum, mean, median abundance and standard deviation of Phylum-level taxa. Abundance measures were produced after removing singleton and taxa present in less than 10% of samples within the dataset.

**Taxa**	**Max relative abundance**	**Mean relative abundance**	**Median relative abundance**	**Standard deviation**
Actinobacteriota	0.9693	0.1159	0.0029	0.2400
Bacteroidota	0.3681	0.0051	0	0.0381
Campilobacterota	0.0054	0.0002	0	0.0007
Firmicutes	1	0.8659	0.9908	0.2571

Fusobacteriota	0.0824	0.0014	0	0.0095
Patescibacteria	7.2767e-05	7.5799e-07	0	7.4268e-06
Proteobacteria	0.4650	0.0113	0.0018	0.0514
Synergistota	0.0086	0.0001	0	0.0009
Verrucomicrobiota	0.0003	4.6013e-06	0	3.6298e-05

Supplementary table S3. Results form nucleotide BLAST alignment. Species-level taxonomic assignment obtained after the alignment of the DNA sequences referred to the two Lactobacillus-assigned ASVs detected by the Wald test of DESeq analysis from different cervix length groups comparison. Nucleotide BLAST assignment and alignment scores were reported.

ASV	Scientific name	Max score	Tot score	Query cover	E value	Perc. Ident.	Acc. Len
ASV_8	*Lactobacillus jensenii*	789	789	100,00%	0.0	100,00%	1500
ASV_12	*Lactobacillus iners*	784	784	100,00%	0.0	99.77%	1539

Supplementary table S4. DNA sequences aligned by using nucleotide BLAST. DNA sequences referred to the two Lactobacillus-assigned ASVs obtained after the DADA2 clustering pipeline and used for the nucleotide BLAST alignment.

ASV	DNA sequence
ASV_8	TAGGGAATCTTCCACAATGGACGAAAGTCTGATGGAGCAACGCCGCGTGAGTGAAGAAGGTT<br>TTCGGATCGTAAAGCTCTGTTGTTGGTGAAGAAGGATAGAGGTAGTAACTGGCCTTTATTTGA<br>CGGTAATCAACCAGAAAGTCACGGCTAACTACGTGCCAGCAGCCGCGGTAATACGTAGGTGG<br>CAAGCGTTGTCCGGATTTATTGGGCGTAAAGCGAGCGCAGGCGGATTGATAAGTCTGATGTGA<br>AAGCCTTCGGCTCAACCGAAGAACTGCATCAGAAACTGTCAATCTTGAGTGCAGAAGAGGAG<br>AGTGGAACTCCATGTGTAGCGGTGGAATGCGTAGATATATGGAAGAACACCAGTGGCGAAGG<br>CGGCTCTCTGGTCTGTAACTGACGCTGAGGCTCGAAAGCATGGGTAGCGAACA
ASV_12	TAGGGAATCTTCCACAATGGACGCAAGTCTGATGGAGCAACGCCGCGTGAGTGAAGAAGGGT<br>TTCGGCTCGTAAAGCTCTGTTGTTGGTGAAGAAGGACAGGGGTAGTAACTGACCTTTGTTTGA<br>CGGTAATCAATTAGAAAGTCACGGCTAACTACGTGCCAGCAGCCGCGGTAATACGTAGGTGG

	CAAGCGTTGTCCGGATTTATTGGGCGTAAAGCGAGTGCAGGCGGTTCGATAAGTCTGATGTGA<br>AAGCCTTCGGCTCAACCGGAGAATTGCATCAGAAACTGTCGAGCTTGAGTACAGAAGAGGAG<br>AGTGGAACTCCATGTGTAGCGGTGAAATGCGTAGATATATGGAAGAACACCGGTGGCGAAGG<br>CGGCTCTCTGGTCTGTTACTGACGCTGAGGCTCGAAAGCATGGGTAGCGAACA

Supplementary table S5. Results from DESeq analysis. Results produced by Wald test from DESeq analysis. Table reported base means (mean of normalized counts) across samples, log2 fold changes, standard error (lfcSE), Wald statistic (stat), Wald test p-value and BH adjusted p-values for each significant ASV with related taxonomic assignment for each taxonomic rank.
